# Decomposition of dinosaurian remains inferred by invertebrate traces on vertebrate bone reveal new insights into Late Jurassic ecology, decay, and climate in western Colorado

**DOI:** 10.7717/peerj.9510

**Published:** 2020-07-15

**Authors:** Julia B. McHugh, Stephanie K. Drumheller, Anja Riedel, Miriam Kane

**Affiliations:** 1 Museums of Western Colorado, Grand Junction, CO, USA; 2 Department of Geology, Colorado Mesa University, Grand Junction, CO, USA; 3 Department of Earth and Planetary Sciences, University of Tennessee, Knoxville, TN, USA

**Keywords:** Paleoecology, Mygatt-Moore Quarry, Ichnology, Dinosaurs, Taphonomy, Jurassic, Residence time, Gastropod, Clerid, Dermestid

## Abstract

A survey of 2,368 vertebrate fossils from the Upper Jurassic Mygatt-Moore Quarry (MMQ) (Morrison Formation, Brushy Basin Member) in western Colorado revealed 2,161 bone surface modifications on 884 specimens. This is the largest, site-wide bone surface modification survey of any Jurassic locality. Traces made by invertebrate actors were common in the assemblage, second in observed frequency after vertebrate bite marks. Invertebrate traces are found on 16.174% of the total surveyed material and comprise 20.148% of all identified traces. Six distinct invertebrate trace types were identified, including pits and furrows, rosettes, two types of bioglyph scrapes, bore holes and chambers. A minimum of four trace makers are indicated by the types, sizes and morphologies of the traces. Potential trace makers are inferred to be dermestid or clerid beetles, gastropods, an unknown necrophagous insect, and an unknown osteophagus insect. Of these, only gastropods are preserved at the site as body fossils. The remaining potential trace makers are part of the hidden paleodiversity from the North American Late Jurassic Period, revealed only through this ichnologic and taphonomic analysis. Site taphonomy suggests variable, but generally slow burial rates that range from months up to 6 years, while invertebrate traces on exposed elements indicate a minimum residence time of five months for carcasses with even few preserved invertebrate traces. These traces provide insight into the paleoecology, paleoclimate, and site formation of the MMQ, especially with regards to residence times of the skeletal remains on the paleolandscape. Comprehensive taphonomic studies, like this survey, are useful in exploring patterns of paleoecology and site formation, but they are also rare in Mesozoic assemblages. Additional work is required to determine if 16.174% is typical of bulk-collected fossils from Jurassic ecosystems in North America, or if the MMQ represents an unusual locality.

## Introduction

Actualistic observations on the decay of vertebrate remains reveal a complex suite of abotic and biotic factors that decompose and modify vertebrate remains (Behrensmeyer, 1978; Behrensmeyer, Kidwell & Gastaldo, 2000; Fisher, 1995). In continental settings, carrion insects play a critical role in the decomposition of soft tissues and, to a lesser extent, skeletal material. Insects and other invertebrates that modify bone (e.g., osteophagus and necrophagous taxa) can leave distinctive traces behind on bone surfaces, providing rare glimpses into the diversity of these rarely-preserved decomposers in the fossil record (Britt, Scheetz & Dangerfield, 2008; Pirrone, Buatois & Bromley, 2014; Roberts, Rogers & Foreman, 2007; Smith, 1986).

The invertebrate faunas in the Upper Jurassic Morrison Formation have received little attention in comparison to their better-known dinosaurian counterparts. Unlike the more prolifically preserved dinosaurian assemblages, invertebrates are rare as body fossils with some groups, like insects, being virtually absent from the fossil record (Gorman et al., 2008; Hasiotis, 2004; Lara et al., 2020; Zherikhin, 2003). Incorporating traces fossils, such as burrows, tracks, borings and feeding marks, has revealed a more complete picture of invertebrate diversity within Late Jurassic ecosystems in North America, and the ichnological record of invertebrate traces made on geological substrates has been documented from sites across the Morrison Formation (Hasiotis, 1999, 2004; Turner & Peterson, 2004). Among previous works examining invertebrate traces, those specifically left on vertebrate bone have received less targeted research.

Here, we present a robust dataset of invertebrate traces on vertebrate fossil material from the Upper Jurassic Mygatt-Moore Quarry (MMQ) in western Colorado. These bioerosion traces are attributable to osteophagus and necrophagous invertebrates, especially insects. These taxa are well-known taphonomic agents in both modern and fossil ecosystems (Behrensmeyer, Kidwell & Gastaldo, 2000; Fisher, 1995), with the oldest known evidence of insect bone surface modifications documented from the Middle Triassic of Brazil (Neto et al., 2016). Although more common in Cretaceous and Cenozoic deposits (Csiki, 2006; Kaiser, 2000; Martin & West, 1995; Paik, 2000; Pirrone, Buatois & González Riga, 2014; Roberts, Rogers & Foreman, 2007; Saneyoshi et al., 2011), insect traces on fossil bone are reported from a few Jurassic localities, including examples from the Morrison Formation of Utah and Wyoming (Bader, 2008; Bader, Hasiotis & Martin, 2009; Britt, Scheetz & Dangerfield, 2008; Chin & Bishop, 2006; Hasiotis et al., 1999). This is the first description of insect traces on fossil bone from the Morrison Formation in Colorado, and the first assemblage-wide survey of invertebrate traces from any Jurassic deposit, providing greater insight into species dynamics of these important decomposers across the paleolandscape than isolated trace descriptions alone.

## Materials and Methods

### Geologic setting

**Figure 1:**
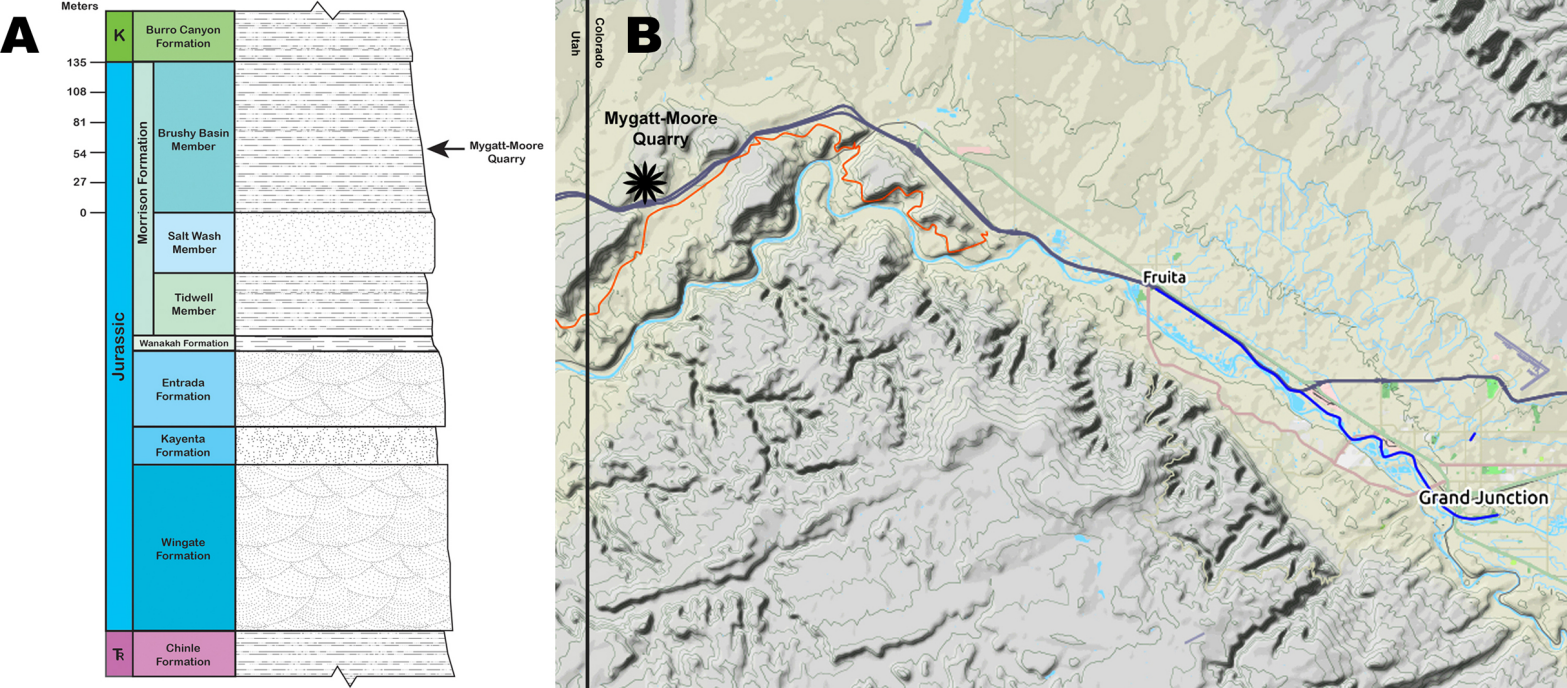
Mygatt-Moore Quarry. (A) Stratigraphic column of the Rabbit Valley area, showing the level of the Mygatt-Moore Quarry. (B) Map of western Colorado showing the location of the Mygatt-Moore Quarry.

The MMQ was discovered in 1981 by J.D. and Vanetta Moore and Pete and Marilyn Mygatt while hiking Rabbit Valley in the McInnis Canyons National Conservation Area, near the Utah-Colorado border (Fig. 1). The site preserves a dinosaur-dominated fossil assemblage within the Brushy Basin Member of the Upper Jurassic Morrison Formation. The Museums of Western Colorado (MWC) began working the site in 1984 and have continued excavations every summer since 1987. The Dinamation International Society worked the site simultaneously with the MWC until 1998. Thousands of vertebrate fossils from the MMQ have been collected and reposited at the MWC, including the holotype specimens of *Hulettia hawesi* (Osteichthyes, Halecostomi) (Kirkland, 1998), *Morrolepis schaefferi* (Osteichthyes, Dipnoi) (Kirkland, 1998) and *Mymoorapelta maysi* (Ornithischia, Ankylosauria) (Kirkland & Carpenter, 1994). Today, the Bureau of Land Management (BLM) co-manages the site with the MWC.

Vertebrate material has been excavated from the MMQ using hand-quarry techniques throughout the site’s history. However, in 2016 all small (minimum of 2.5 cm^2^), unidentifiable bone fragments that preserved cortical tissue were collected in 5-gallon buckets, termed “nugget buckets”, explicitly for analysis in this survey of bone surface modifications. Prior to 2016, these small fragments were systematically not collected, and reburied at the site without study. Whole site collection was essential to this survey in order to understand the extent of trace markings throughout the assemblage (Drumheller et al., 2020). Collection of all vertebrate fossil material at the MMQ by the authors was carried out under BLM paleontological permit number COC76588.

The main horizon at the MMQ is a 1–2 m-thick unit that varies from laminated to medium bedded gray silty mudstones that lies atop a layer of micritic pebbles and is stratigraphically located 64 m above the base of the Brushy Basin Member (Foster, 2003, 2014; Foster et al., 2018; Foster & Hunt-Foster, 2011; Kirkland & Carpenter, 1994; Mygatt, 1991) (Fig. 1). The transition from laminated to medium bedding is sporadic and occurs in isolated pockets, likely from bioturbation events by the Jurassic vertebrate fauna. Dinosaurian remains are common throughout the main horizon, but are concentrated in the lower beds. Molluscan fauna and microvertebrates (e.g., amphibians and rhynchocephalians) are found only in the upper 50 cm of the main horizon. Radiometric analysis of ash-fall zircons from the quarry returned an age of 152.18 ± 0.29 Ma for the site (Trujillo et al., 2014).

The MMQ is interpreted to preserve a riparian ecosystem, with abundant vegetation and a high water table, but without perennial standing water, similar to modern-day watering holes, bogs, and mires (Foster, 2003, 2014; Foster et al., 2018; McHugh, 2018; McHugh et al., 2018; Mygatt, 1991; Trujillo et al., 2014). Carbonized plant material is prolific within the quarry, however aquatic vertebrates such as crocodylomorphs, turtles, and fish are rare in the main horizon, supporting the interpretation that there was no continual standing water in the ecosystem, though in the limestone horizon (“fish layer”) of the quarry (stratigraphically above the dinosaur-bearing main horizon) this changes (Foster, 2014; Foster et al., 2018). Previous taphonomic work established this as an autochthonous assemblage within an attritional overbank environment with seasonal deposition, resulting in few articulated specimens and no preferred orientation of skeletal elements (Foster, 2014; Foster et al., 2018).

### Bioerosion trace identification

We surveyed 2,368 fossil specimens from the MMQ, which in early 2020 included all vertebrate material, excluding teeth, that was not still under preparation, on exhibit, or out on loan (Table 1). We examined fossil material for bioerosion traces using raking light and low magnification, following the methodology of Blumenschine, Marean & Capaldo (1996). Higher magnification was used to assess morphologies of identified traces. We identified multiple types of bioerosion in the MMQ assemblage, including vertebrate bite marks, previously described by Drumheller et al. (2020), and a diverse assemblage of invertebrate traces, including bore holes, chambers, rosettes, pits and furrows and bioglyph scrapes (Table 2). These traces were measured using digital calipers (length vs. width) and identified and described based on the ichnotaxobases of Pirrone, Buatois & Bromley (2014). We then compared trace morphologies with those known from other fossil sites, as well as modern analogs, to infer both trace behavior and trace maker.

**Table 1:** Examined Materials. Examined materials from the Mygatt-Moore Quarry categorized by taxonomic group and presence/absence of bone surface modifications.

Taxon	Invertebrate traced bones	Bones with any BSM	Bones examined	Invertebrate traces (%)	Total BSM (%)
Sauropoda	305	582	1,064	28.665	54.699
Theropoda	44	105	428	10.280	24.533
*Mymoorapelta*	5	28	174	2.874	16.092
Other tetrapods	28	110	300	9.333	36.667
“Nugget buckets”	1	59	402	0.249	14.677
Total	383	884	2368	16.174	37.331

**Table 2:** Bone Surface Modifications. Types of bone surface modifications found in the Mygatt-Moore assemblage. Numerous elements preserved multiple types of traces; therefore, this is a tabulation of all individual traces, not individual bone elements. Subtotals for larger bone surfaces modification categories (i.e., bite marks, invertebrate traces, and other) are denoted in grey cells and bold text.

	Theropod material	Sauropod material	*Mymoorapelta maysi*	Other tetrapods	“Nugget buckets”	Total marks	Percent marked
Bite marks	**260**	**1,060**	**31**	**97**	**61**	**1,509**	**69.893**
Edge marks	1	0	0	0	0	1	0.049
Furrows	6	22	0	6	0	34	1.658
Pits	27	40	9	13	1	90	4.388
Serial pits	5	1	0	0	0	6	0.293
Punctures	12	18	1	3	1	35	1.706
Scores	175	877	20	67	55	1,194	58.216
Serial scores	19	53	0	1	4	77	3.754
Striations/striated scores	16	45	1	6	0	68	3.315
Striated furrows	0	4	0	1	0	5	0.244
Invertebrate traces	**61**	**340**	**5**	**28**	**1**	**435**	**20.148**
Pits/furrows	61	323	5	28	1	418	20.380
Bore holes/chambers	0	12	0	0	0	12	0.585
Bioglyph Scrapes	0	5	0	0	0	5	0.244
Other marks	**24**	**172**	**0**	**12**	**7**	**215**	**9.958**
Abrasion	2	5	0	1	0	8	0.390
Depressions	3	36	0	1	1	41	1.999
Etching	0	4	0	1	0	5	0.244
Fractures	3	3	0	1	0	7	0.341
Prep damage	5	11	0	5	0	21	1.024
Root marks	11	108	0	3	6	128	6.241
Other/unknowns	0	5	0	0	0	5	0.244

## Results

### Insect trace frequency

Of the 2,368 surveyed fossils from the MMQ, 884 specimens preserved some type of bone surface modification (BSM), representing 37.331% of the examined materials (Table 1; Tables S1 and S2). Many fossils preserved multiple marks and multiple types of marks. The majority of the 2,161 observed marks on the 884 specimens were feeding traces from theropod dinosaurs and other vertebrate actors (69.829% of total BSM, 28.927% of total assemblage) (Table 2) (Drumheller et al., 2020). Traces left by invertebrates were the second most common type of BSM, found on 383 specimens (43.326% of the modified materials, 16.174% of total assemblage), and include 435 individual invertebrate traces (20.222% of total BSMs) and six discrete trace types that are described below.

Taxonomic tabulations of these traces showed the vast majority (78.261%) of all invertebrate traces occur on sauropod fossil material (342 individual traces). This could potentially reflect the large size of these animals and their proportionately longer exposure times in an attritional overbank depositional environment, but the frequency at which they are marked aligns with the overarching taxonomic composition of the site (Foster, 2014; Foster et al., 2018). Theropod material, the second largest taxonomic group found at MMQ, contained 13.956% of the observed insect traces. The remaining trace percentages include *Mymoorapelta maysi* (1.144%), other tetrapods (6.407%), and isolated bone fragments (0.229%).

### Invertebrate trace types

#### Trace type 1—pits and furrows

**Figure 2:**
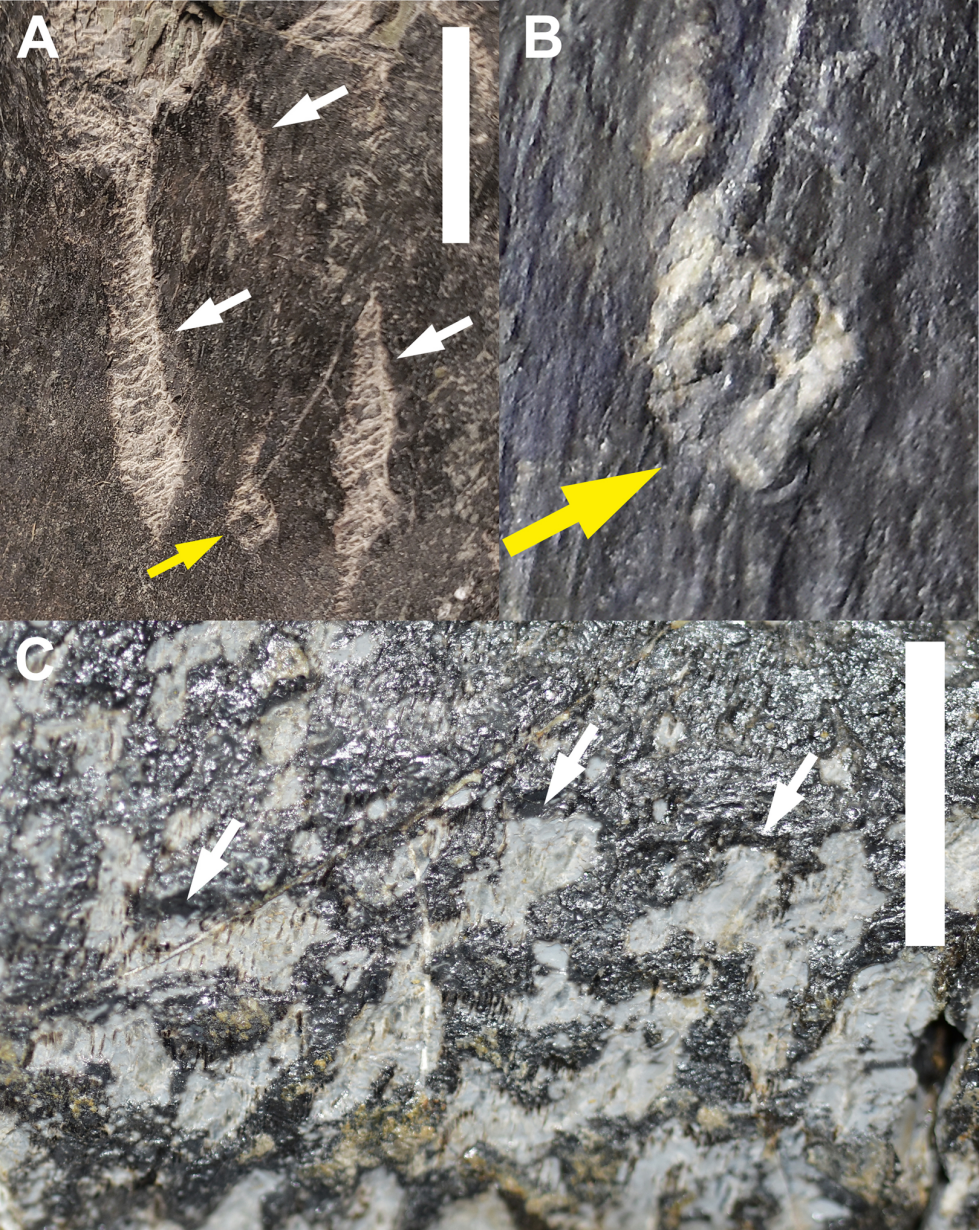
Trace Type 1. (A) White arrows indicate several furrows of *Cubiculum ornatus* on a sauropod rib (MWC 9032) and yellow arrow indicates a rosette (Trace Type 2); (B) higher magnification photo of the rosette in 2A, indicated by yellow arrow; (C) white arrows indicate ornamented and unornamented furrows of *C. ornatus* on a sauropod pubis (MWC 9559). All scale bars equal 10 mm.

The most common and widespread invertebrate traces within the MMQ fossil assemblage are groupings of shallow pits and furrows that often, but not always, include bioglyphs. Bioglyphs are carvings or scratchings on bone surfaces that were generated by necrophagus or osteophagus activities (Ekdale & De Gibert, 2010); they are important ichnotaxobases for interpreting trace formation (Pirrone, Buatois & Bromley, 2014). These groupings of shallow pits and furrows are characterized by their concave walls and haphazard arrangements along the surfaces of bones. These traces are identified as *Cubiculum ornatus* (Roberts, Rogers & Foreman, 2007) based on their depth, concave wall structures, and associated bioglyphs; they represent 95.652% of all identified invertebrate traces in the dataset (Table 2). *Cubiculum ornatus* are pits and furrows that are not backfilled and are present through the assemblage as either isolated groupings or dense clusters of pits and furrows that overlap each other. These traces preserve mandibular scrapes as bioglyphs within the pits and furrows (Fig. 2A). These biogylphs can be distinctive, faint, or absent, even within adjacent furrows (Fig. 2C).

The biogylphs in *C. ornatus* present as arcs sweeping toward the center of the furrow. These arcs can be isolated scrapes or joined to form a single arc with the apex pointed in single direction throughout the structure, presumably in the opposite direction of the actor’s movement. These types of biogylphs have been attributed to the mandibular elements of insects (Roberts, Rogers & Foreman, 2007). In the MMQ assemblage, *C. ornatus* are found on theropod, sauropod, ornithischian, and indeterminate dinosaur material, as well as isolated bone fragments collected in “nugget buckets” (Table 2).

#### Trace type 2—rosettes

A sauropod proximal rib fragment (MWC 9032) preserves numerous clusters of Trace Type 1, but on the anterior of the rib head, among the *Cubiculum ornatus* traces, are small and distinct rosettes (Figs. 2A and 2B). These rosettes are subcircular in shape, with an outer rim of excavated periosteum and a scalloped margin surrounding a pedestal of surface bone in the center. These traces are very small, averaging 2.436 mm in diameter, with the inner pedestals averaging 1.159 mm in diameter. Bioglyphs are present and contained entirely within the broken periosteum of the rosette, as in Trace Type 1, and are absent from the remaining periosteum on the inner pedestal, indicating the rosettes were formed in a circular array of ostephagy by a small actor and not in a single event by a large actor. Bioglyphs also do not radiate away into the surrounding bone surface, forming the diagnostic “star” bioglyphs identified in actualistic studies using termites (Backwell et al., 2012). These “star” shapes are absent in all identified rosettes found in this study.

#### Trace type 3—bore holes

**Figure 3:**
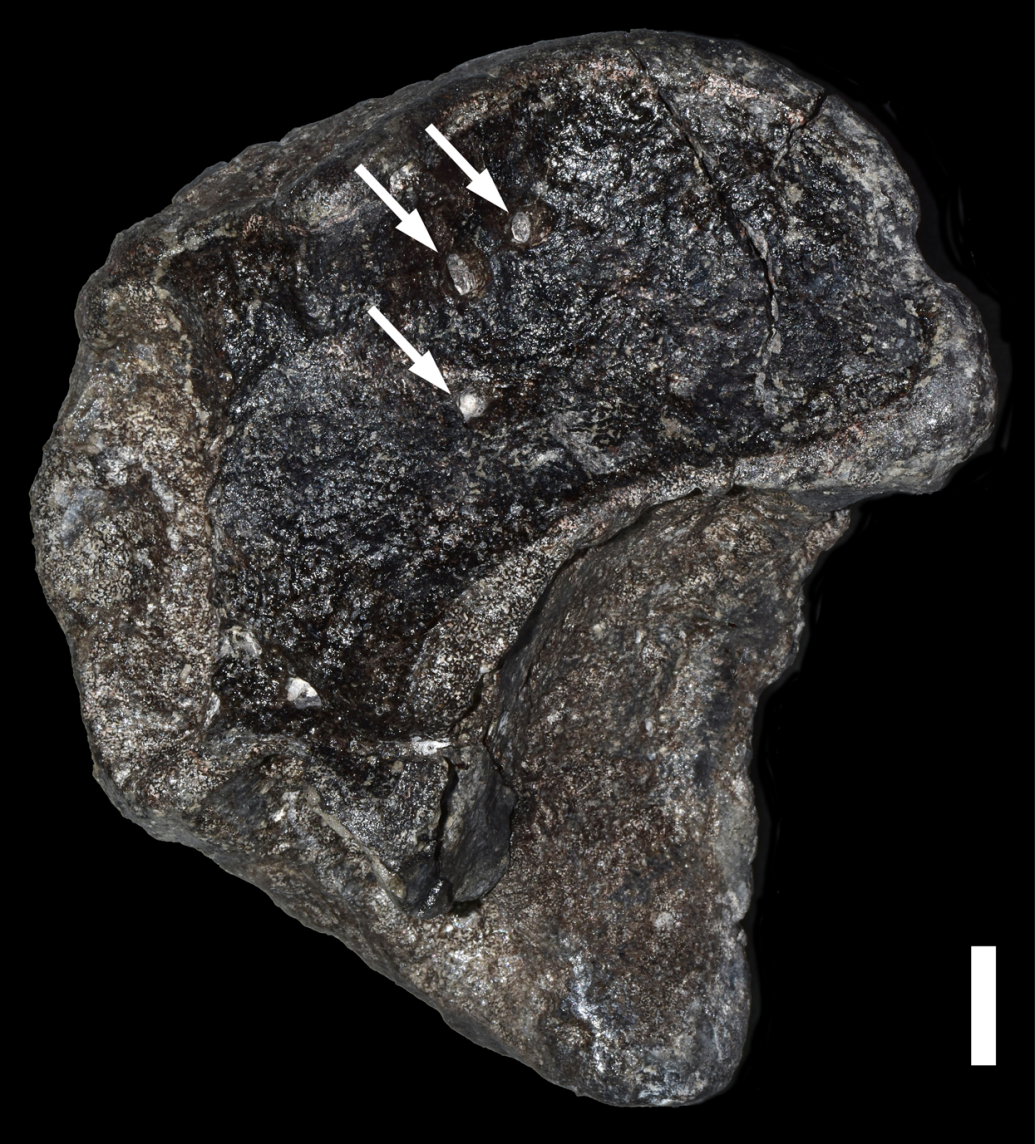
Trace Type 3. White arrows indicate small bore holes in a sauropod condylar fragment (MWC 8968). Scale bar equals 10 mm.

Numerous bore holes were identified on a single fragment of sauropod material. Ten small bore holes occur in two clusters on opposite faces of a sauropod condylar fragment (Fig. 3; Table 2). These holes are subcircular, with most measuring 1–3 mm in diameter. Each hole shallowly penetrates the periosteum as a hollow cylinder, but does not change direction or proceed at great depth, as is common with tunnels. No bioglyph scrapes were identified in the hole walls or around the openings, as is often found with holes made by termites (Backwell et al., 2012).

#### Trace type 4—chambers

**Figure 4:**
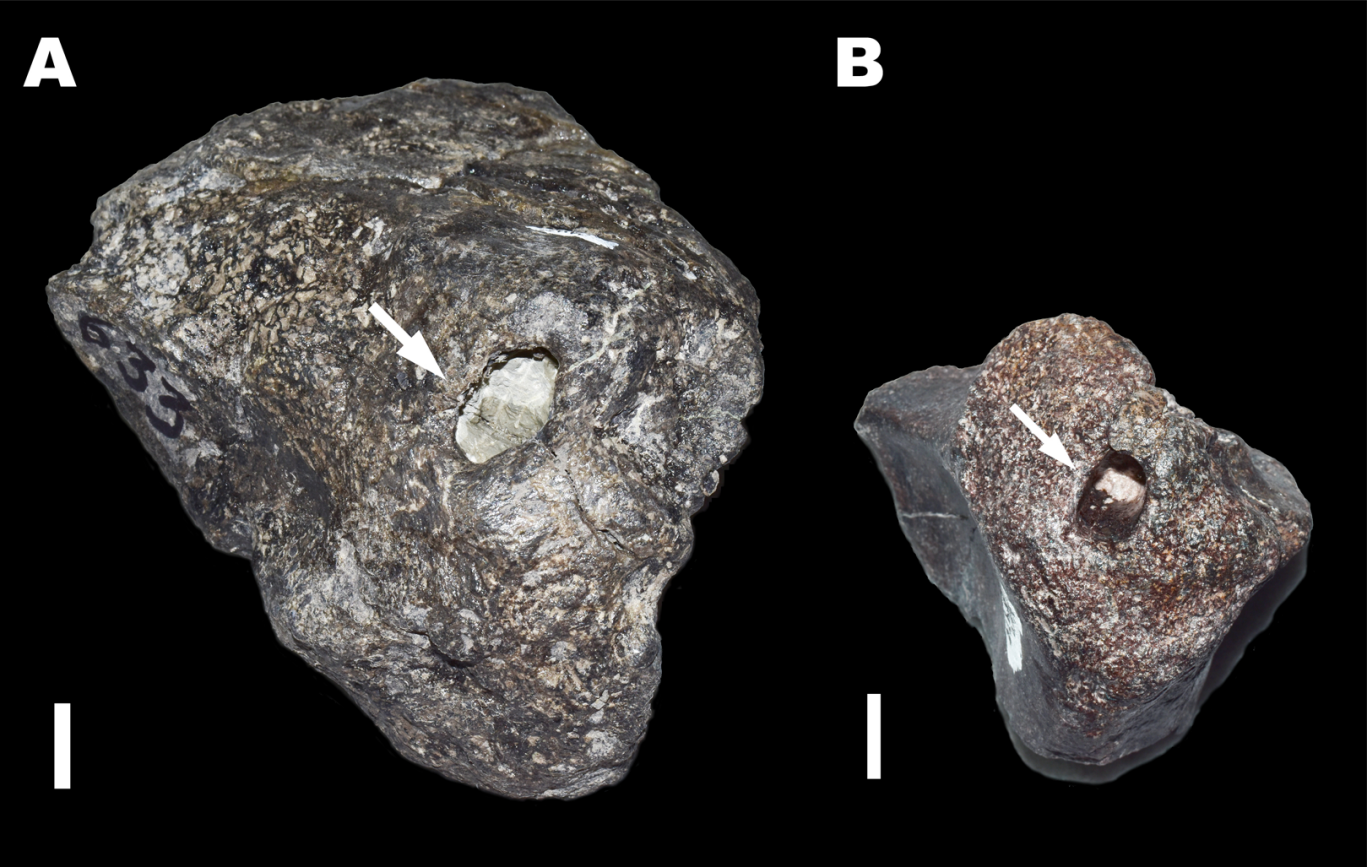
Trace Type 4. White arrows indicate large ovoid chambers on sauropod bone fragments (A: MWC 8975, and B: MWC 9228). All scale bars equal 10 mm.

Large and deep ovoid chambers were identified on two separate bone fragments (Fig. 4; Table 2). These chambers are an order of magnitude larger than those described in Trace Type 3, measuring 1.15–1.51 cm in length and 0.79–0.88 cm in width. Chamber entrance margins are smooth on fragment MWC 8975 and irregular on MWC 9228. Neither fragment preserves bioglyph scrapes at the margins or inside their chambers, making actor identification difficult. The size discrepancy between these chambers and the smaller bore holes in Trace Type 3, indicates different actors were involved in the formation of these two trace types. Excavation of these chambers would have required osteophagy and the removal of bone material rather than its redeposition, as no infilling is present in either specimen. These chambers contain remnants of matrix from the quarry site, and not biologically created fillings, suggesting they were left open by the actor or filled with a secreted substance that deteriorated prior to fossilization (Paik, 2000; Pirrone, Buatois & Bromley, 2014).

#### Trace type 5—punctuated biogylphs

**Figure 5:**
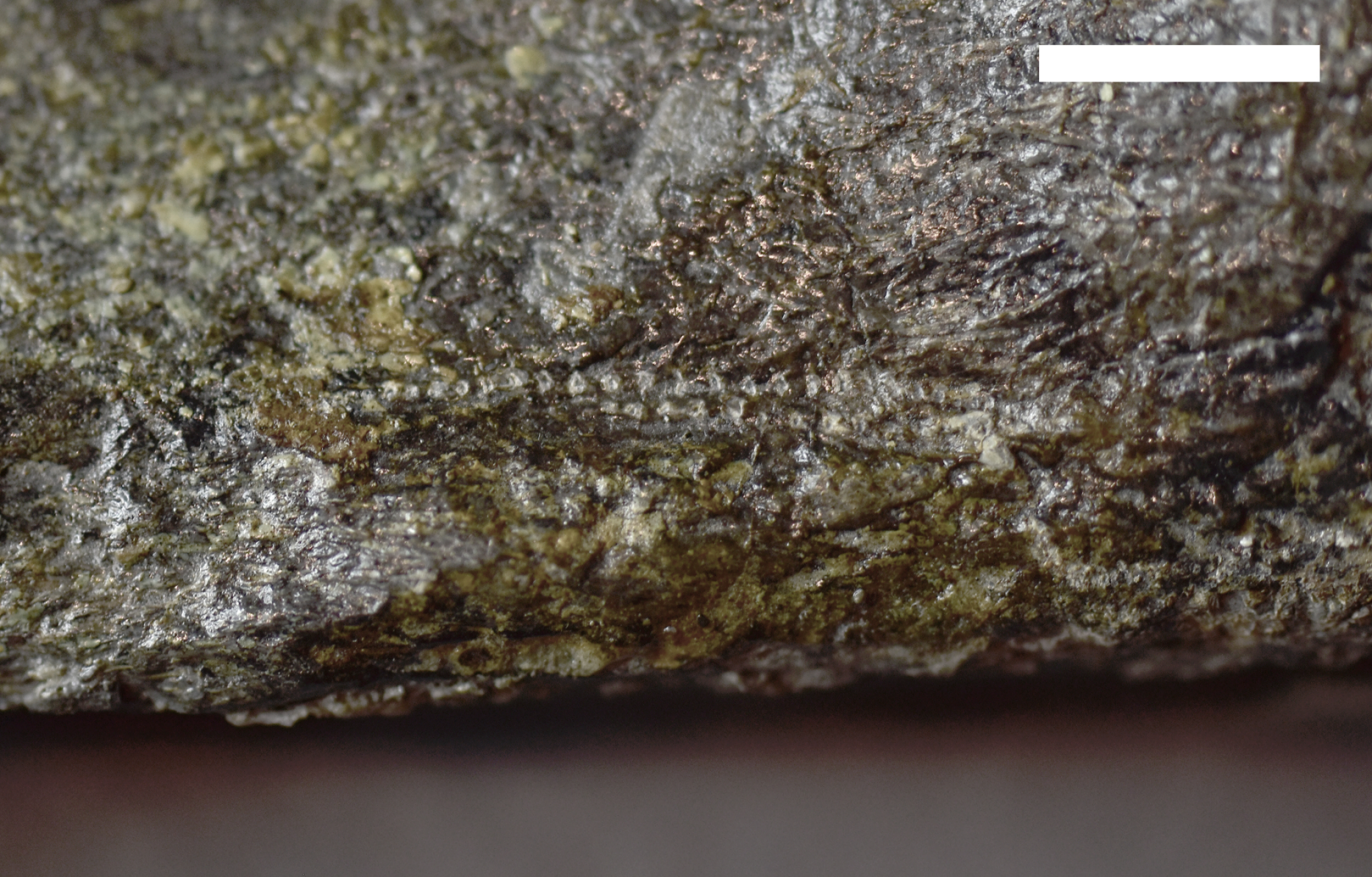
Trace Type 5. *Machichnus bohemicus* preserved on a sauropod bone fragment (MWC 316). Scale bar equals 10 mm.

Five specimens preserve lines of punctuated bioglyph scrapes without any periosteum excavation. This biogylph pattern is named *Machichnus bohemicus* (Mikuláš et al., 2006) (Fig. 5) and is characterized by series of subparallel, paired bioglyphs on the periosteum. Each individual scrape is very short and does not overlap or approach any adjacent scrape. These individual marks average 0.577 mm in length. Each pair is approximately one millimeter apart, penetrating shallowly into the cortical tissue, and continue for 10–15 mm in total trace length. These patterns are markedly straight and do not show the haphazard or wandering placement that is common in Trace Type 1 or Trace Type 6 (see description below). The paired bioglyphs of *M. bohemicus* do not connect at the midline or to the next pair in the sequence. These distinct, short linear scrapes are the result of mandibular strikes on the periosteum (Mikuláš et al., 2006), but do not conjoin to form a complete pit or furrow as in Trace Type 1, rather these do not represent directed manipulation of bone material in osteophagy, but appear to be the result of necrophagy, the feeding on the unpreserved, overlying soft tissues and sinews.

#### Trace type 6—meandering biogylphs

**Figure 6:**
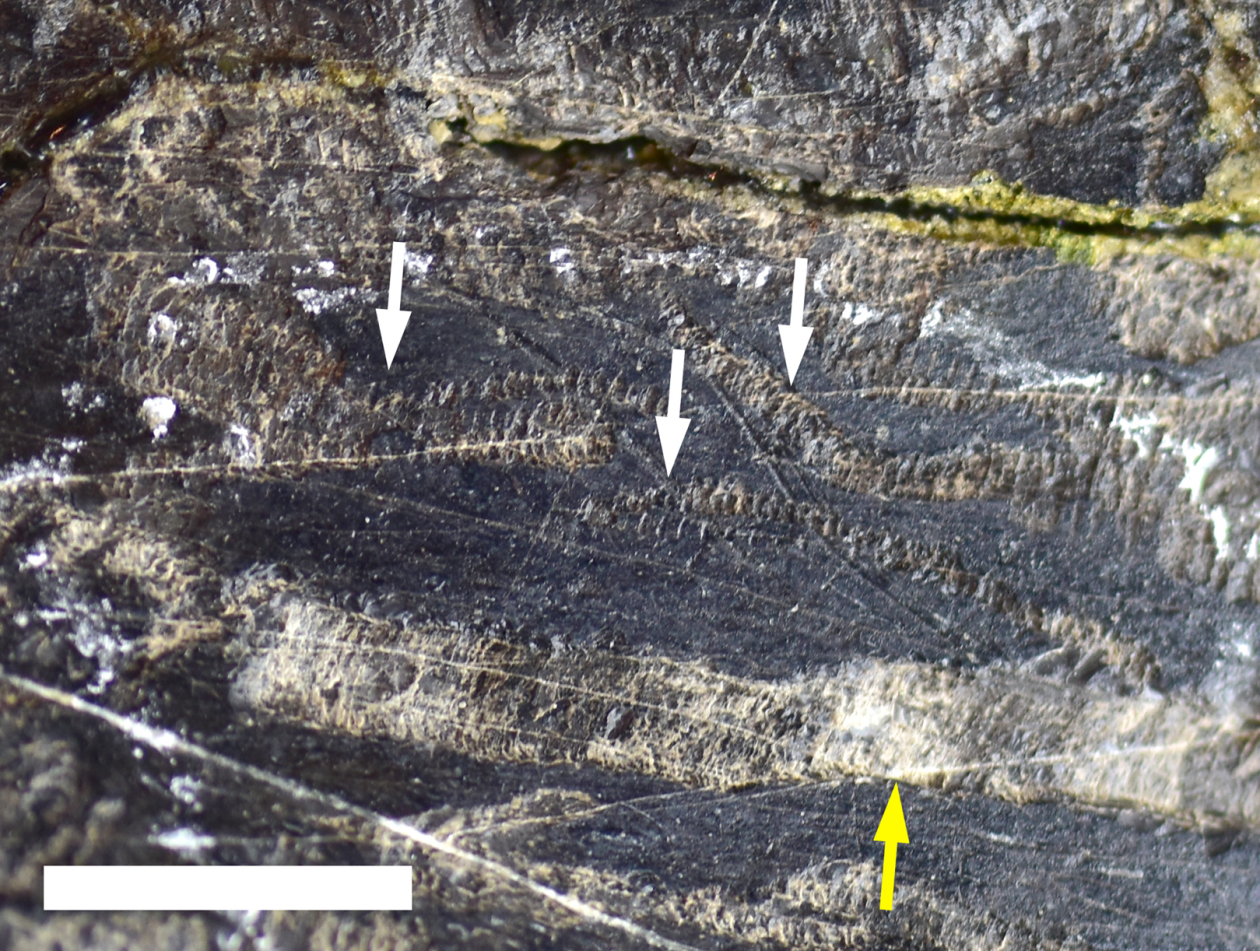
Trace Type 6. White arrows indicate several trails of *Osteocallis mandibulus* on the surface of a sauropod rib fragment (MWC 9032). Yellow arrow indicates a furrow of *Cubiculum ornatus* (Trace Type 1). Scale bar equals 10 mm.

Multiple meandering trails of bioglyph scrapes in close proximity to each other and without any periosteum excavation were identified on the posterior margin of MWC 9032, a proximal sauropod rib fragment (Fig. 6). Unlike the bioglyph scrapes in Trace Type 5, these traces combine to form sinuous trails along the bone surface. These trails are identified as belonging to the ichnotaxon *Osteocallis mandibulus* (Roberts, Rogers & Foreman, 2007), which is a series of arcuate scrapes that extend for centimeters along the bone surface, which can often overlap and connect at the midline and form a meandering trail along the periosteum. Ranging in width from 0.967 to 2.880 mm, these traces proceed for 10 s of cm along the surface of the bone without extensive excavation into the underlying periosteum. These necorphagus traces were found in close proximity to several excavated furrows (osteophagy) identified as *Cubiculum ornatus* traces (Trace Type 1).

## Discussion

### Potential actors

Bioerosion traces are artifacts of behavior, with many species able to leave different types of traces. The different traces made can reflect changes in substrate, morphology, behavior, or ontogeny. Additionally, separate species are capable of creating the same traces due to similarities in behavior, ontogeny, morphology or substrate, a complicating factor when identifying convergent taphonomic modifications called equifinality (Behrensmeyer, Kidwell & Gastaldo, 2000). We identified six types of invertebrate traces in the Mygatt-Moore fossil assemblage and their associated ichnotaxa. Insects known for modifying bone at various stages of ontogeny include dermestid and clerid beetles, termites, tineid moths, mayflies, some species of solitary wasps and bees, and some species of ants (Backwell et al., 2012; Britt, Scheetz & Dangerfield, 2008; Deyrup et al., 2005; Fernández-Jalvo & Monfort, 2008; Freymann et al., 2007; Holden, Harris & Timm, 2013; Pittoni, 2009; Watson, 1986; Zanetti, Visciarelli & Centeno, 2014, 2015). Other terrestrial and freshwater invertebrates known to modify vertebrate bone are carnivorous gastropods and burrowing bivalves (Janssen, Van Baal & Schulp, 2013; Serrano-Brañas, Espinosa-Chávez & Maccracken, 2018; Tapanila et al., 2004) (Table 3). We can exclude any putative marine actors, as the Brushy Basin of western Colorado is well-supported as a freshwater fluvial system (Foster et al., 2018, Maidment & Muxworthy, 2019; Turner & Peterson, 2004). Our working hypotheses for putative actors for each identified trace type are summarized in Table 4.

**Table 3:** Potential vertebrate and invertebrate trace makers cross-referenced with the evidence for or against their assignment as an actor. X indicates that observed trace is inconsistent with the known sizes and/or trace morphologies made by the actor; √ indicates that the observed trace is consistent with the observed size and morphology of the actor; √√ indicates that the observed trace is both consistent with the size and morphology of the actor and has been observed to be made by the actor in live colony experiments; ? indicates traces morphologies not observed in the dataset.

Potential actor	Trace morphology	First occurrence	Found at MMQ	Trace type 1	Trace type 2	Trace type 3	Trace type 4	Trace type 5	Trace type 6
Vertebrates									
Mammals	Gnawing at epiphyses, margins & broken edges	Middle Jurassic	No	X	X	X	X	X	X
Salamanders	Unknown osteophagia	Early Jurassic	Yes	X	X	X	X	X	X
Pleurosternid Turtles	Breakage	Middle Jurassic	Yes	X	X	X	X	X	X
Rhynchocephalians	Unknown osteophagia	Early Triassic	Yes	X	X	X	X	X	X
Mollusks									
Burrowing Bivalves	Clavate-shaped borings	Cambrian	No	X	X	X	X	X	X
*Viviparus* or *Amplovalvata*	Small and shallow subcircular borings	Middle Jurassic	Yes	X	X	√	X	X	X
Insects									
Dermestid larvae	Shallow, isolated or overlapping furrows and chambers	Late Triassic	No	√√	√√	√	√	X	X
Dermestid adults	Shallow, isolated or overlapping furrows and chambers	Late Triassic	No	X	X	X	X	X	√√
Clerid larvae	Shallow, isolated or overlapping furrows and chambers	Middle Jurassic	No	√√	√√	√	√	X	X
Clerid adults	Shallow, isolated or overlapping furrows and chambers	Middle Jurassic	No	X	X	X	X	X	√√
Termites	Star biogylphs, complex nests and chambers	Eocene	No	X	X	X	X	X	X
Tineid Moth larvae	Isolated chambers in keratinous structures	Eocene	No	?	X	X	X	X	X
*Odontotermes* larvae	Isolated chambers in keratinous structures	Extant	No	?	X	X	X	X	X
Mayfly larvae	U-shaped chambers	Middle Triassic	No	X	X	X	X	X	X
Ants	Complex networks, nests, and chambers	Late Cretaceous	No	X	X	X	X	X	X
Burrowing Wasps	Irregular shaped borings 2-20 mm in diameter	Late Cretaceous	No	X	X	X	√	X	X
Burrowing Bees	Irregular shaped borings 2-20 mm in diameter	Eocene	No	X	X	X	√	X	X

**Table 4:** Working hypotheses of potential actors, ontogenetic stages, and associated traces. Unassigned working hypotheses for given traces are indicated with “—”.

	Trace type 1	Trace type 2	Trace type 3	Trace type 4	Trace type 5	Trace type 6
Working hypothesis 1	Dermestid larvae	Dermestid larvae	Gastropods	Unknown taxon	Unknown taxon	Adult dermestids
Working hypothesis 2	Clerid larvae	Clerid larvae	Dermestid larvae	Oversized dermestid larvae	—	Adult clerids
Working hypothesis 3	Unknown taxon	Unknown taxon	Clerid larvae	Oversized clerid larvae	—	Unknown taxon
Working hypothesis 4	—	—	Unknown taxon	—	—	—

Among the examined material, no identified traces are consistent with the sophisticated network-chamber systems created by social insects (e.g., Formicidae and Termitoidae), who build complex nests as opposed to the isolated chambers observed here. We were also not able to identify any star bioglyphs, which have been identified in experimental studies as diagnostic traces made by bone-modifying termites (Backwell et al., 2012). Based on these absences among the observed traces, we cannot positively refer any traces to termites or ants as potential actors in this dataset. Tineid moths and the termite genus *Odontotermes* create their pupation chambers in keratin and can impact the underlying bone cores (Deyrup et al., 2005; Freymann et al., 2007). One taxon in this dataset, *Mymoorapelta maysi*, is an armored ankylosaur with numerous keratin-covered osteoderms; yet none of the osteoderms preserved any bore holes or chambers. Neither did any of the observed ungals of other taxa. This suggests that tineid moths and *Odontotermes* are likely not potential actors to the bore holes and chambers observed here. Mayfly larvae can also be excluded as potential actors, as their pupation chambers are characteristically “U-shaped” (Britt, Scheetz & Dangerfield, 2008; Xing et al., 2013), which are not observed in any of the Mygatt-Moore material. Pittoni (2009) observed in an archaeological context chamber formation on bone material consistent with the size and morphology of the ovoid chambers found at MMQ in Trace Type 4. These chambers were the result of burrowing by species of solitary wasps and bees (Sphecidae and Halicitidae). However, these lineages do not appear in the Jurassic; the oldest fossil occurrence of Sphecidae is in the Cretaceous and Halictidae is in the Eocene, with molecular studies supporting a Late Cretaceous divergence for the two clades (Branstetter et al., 2017), which eliminates these two clades as possible trace makers for Trace Type 4. This leaves members of Coleoptera (Dermestidae and Cleridae) and any putative unknown, but behaviorally convergent, fossil taxa as potential insect actors at the site. The oldest fossil clerids are from the middle to late Jurassic of China (Wang, Zhang & Fang, 2006) and the oldest non-controversial body fossils of dermestids are from the Middle Jurassic of China (Deng et al., 2017), although the precise age of this site is disputed, which places their clades temporally within the current dataset.

Of the six described invertebrate traces, several ichnotaxa and trace types have been identified elsewhere in Morrison Formation deposits, including rosettes, *Osteocallis mandibulus*, *Cubiculum ornatus*, chambers and bore holes (Bader, 2008; Bader, Hasiotis & Martin, 2009; Britt, Scheetz & Dangerfield, 2008; Hasiotis et al., 1999). Although the validity of some ichnofossil identifications have been disputed, particularly those requiring extensive ghost lineage extensions (Grimaldi & Engel, 2005), the reported Morrison traces are consistent with those observed in actualistic studies using dermestid beetles and have been inferred in the paleontological literature to be the work of dermestids (Bader, Hasiotis & Martin, 2009). However, as invertebrate traces on vertebrate bone from the Jurassic Period are rare, as well as insect body fossils from this time period, we cannot rule out the possibility of an unknown “ghost” taxon not yet described from the fossil record as a potential actor in this dataset.

Three of the identified trace types were found together, and in close proximity, on a single fossil specimen: *Cubiculum ornatus, Osteocallis mandibulus* and rosettes. All three of these traces have been positively referred to dermestid beetles by other authors (Bader, Hasiotis & Martin, 2009; Britt, Scheetz & Dangerfield, 2008). Roberts, Rogers & Foreman (2007) also identified *C. ornatus* and *O. mandibulus* in close association on vertebrate bone material from Late Cretaceous deposits in Utah and Madagascar. They interpreted *O. mandibulus* as a feeding trace and *C. ornatus* as a pupation trace and suggested the two traces may have been formed by the same actor in different behavioral modes or at different ontogenetic stages. The co-occurrence of these traces in the Mygatt-Moore assemblage, and even on the same specimen, support this interpretation. Bader, Hasiotis & Martin (2009), using a dermestid colony, determined that the rosettes are an early stage in the production of pupation pits by dermestid beetles, which is supported by their presence alongside the more developed pupation pits of *C. ornatus* in this fossil.

The *Machichnus bohemicus* traces identified in this dataset (Fig. 5) differ from those in more recent fossil assemblages in that they are short, percussive scrapes that do not form the long sweeping arcs of gnawing rodents or feeding sharks (Mikuláš et al., 2006; Milan, Lindow & Lauridsen, 2011). Instead, they are a subparallel series of paired scrapes likely generated by the same type of feeding behavior in a different taxon. Unlike the bioglyphs preserved in *O. mandibulus* and *C. ornatus*, these scraps are straight, not arcuate, and do not meet or even approach each other at the midline. Thus, it is unlikely that they are the result of the same mandibular apparatuses. Although this trace is known to be generated by vertebrate taxa, such as rodents, their traces are substantially longer and wider, form arcs, tend to overlap, and are often positioned on bone margins (Mikuláš et al., 2006; Milan, Lindow & Lauridsen, 2011), whereas the traces observed here are not. Additionally, Mesozoic mammals are absent from the MMQ fossil assemblage (Foster et al., 2018), although they are found at quarry sites in the surrounding region (Davis, Cifelli & Rougier, 2018; Engelmann & Callison, 1999; Hughes et al., 2015; Luo & Wible, 2005; Rasmussen & Callison, 1981). Mesozoic mammals include many groups unrelated to modern rodents (e.g., docodonts, multituberculates) and tend to have paired incisors with fluted enamel, which have been shown to more deeply penetrate the periosteum, leaving subscores within the traces that can often form overlapping sets (Kielan-Jaworowska, Cifelli & Luo, 2005; Klippel & Synstelien, 2007; Longrich & Ryan, 2010). These traces are inconsistent with the bioglyph patterns reported here. Thus, we can reject mammals as putative trace makers.

The *M. bohemicus* traces are also inconsistent with known bite mark morphologies from theropods and crocodylians (D’Amore & Blumensehine, 2009; Drumheller & Brochu, 2014, 2016; Jacobsen & Bromley, 2009; Njau & Blumenschine, 2006) including those specifically known from the MMQ (Drumheller et al., 2020), and are incompatible with size and tooth morphology of sauropods and ornithischians. Other possible vertebrate actors in the Mygatt-Moore assemblage include turtles, salamanders and rhynchocephalians (Foster et al., 2018). In modern observational studies, turtles have been noted to use their keratinous beaks to pry off chips of weathered bone for ingestion, rather than percussively gnawing on the bone surface (Esque & Peters, 1994), which is inconsistent with the morphology of *M. bohemicus*. As for amphibians, the anterior tooth morphology of salamanders is not compatible with the protruding incisors needed to create this trace, and adult rhynchocephalians are too large to be a potential actor. Juvenile rhynchocephalians may be of adequate size to make this trace, but osteophagy has never been reported in this group. Also, as rhynchocephalian teeth fuze to the jaws during ontogeny and very young animals may be incapable of osteophagy. Therefore, we can disregard juvenile rhynchocephalians as potential actors.

In the absence of a plausible vertebrate actor, we look to the invertebrate fauna. The paired pattern in *M. bohemicus* is inconsistent with gastropod and bivalve anatomy. Some insects possess the paired mandibular structures required to make paired traces like these. However, the actor is displaying a markedly different feeding pattern, one that is much more percussive and linear than those preserved in *O. mandibulus* and *C. ornatus*, indicating a separate necrophagous taxon as the potential actor. Necrophagous insects, such as adult dermestid or clerid beetles or functionally convergent taxa, would also be of consistent size to create this trace with mandibular strikes within the periosteum (Bader, Hasiotis & Martin, 2009; Britt, Scheetz & Dangerfield, 2008; Roberts, Rogers & Foreman, 2007).

Reports of boring activity similar to those described in Trace Type 3 come from the Morrison Formation of Wyoming, and have been exclusively attributed to Jurassic relative of modern dermestid beetles based on their morphologies and associated biogylphs (Britt, Scheetz & Dangerfield, 2008; Hasiotis et al., 1999; Höpner & Bertling, 2017; Kirkland & Bader, 2010). The circular bore holes and ovoid chambers identified in this study (Trace Types 3–4) could be the result of dermestid or other beetles, as have been inferred from other Mesozoic specimens, but carnivorous gastropods and burrowing bivalves can also create bore holes in vertebrate bone (Janssen, Van Baal & Schulp, 2013; Serrano-Brañas, Espinosa-Chávez & Maccracken, 2018; Tapanila et al., 2004). Bore holes of the size and morphology observed in Trace Type 3 are consistent with those created by both dermestid beetles and carnivorous gastropods. Two gastropod genera are known from the MMQ: the omnivore *Viviparus* and the carnivore *Amplovalvata*. (Foster et al., 2018; Mygatt, 1991). The borings of carnivorous gastropods tend to be round to subcircular, which is consistent with the small bore holes identified in Trace Type 3. The size of the bore holes is also consistent with the size of fossil gastropods found at the quarry. Still, without discernable bioglyphs we cannot positively refer either gastropods, bivalves, or insects as an actor for Trace Type 3, but each taxon could reasonably be postulated as potential actors.

As for the deep, ovoid chambers of Trace Type 4, these holes are much larger than Trace Type 3 and exceed the possible size range of the MMQ gastropod fauna. In marine and near-shore deposits, bivalves have been reported to create bore holes of this size in bone (Tapanila et al., 2004); however, the hole openings are circular or clavate, not ovoid. Because of this and the fact that bivalves are not found in the MMQ fauna, we are eliminating them as possible trace makers for Trace Type 4. The size and morphology of the chambers are consistent with those found in archaeological settings, created by solitary wasps and wild bees (Pittoni, 2009). However, the clades containing these insects (Sphecidae and Halicitidae) are not found in Jurassic deposits, and instead have origins in the Late Cretaceous Period, which is supported by both fossil evidence and molecular divergence dates (Branstetter et al., 2017). Thus, we are inferring these traces as originating from insects that exhibited convergently similar bone-modifying behavior to extant Sphecidae and Halicitidae, but are different taxa from these and those involved in Trace Type 3, or alternatively, may represent different ontogenetic stages of a single taxon that was putatively involved in Trace Type 3.

### Hidden paleodiversity

We infer at least four potential actors within this dataset, including dermestid beetles (rosettes, *C. ornatus*, *O. mandibulus*), a large unknown osteophagus insect (large ovoid chambers), gastropods or coleopterans (small bore holes), and an unknown necrophagous insect (*M. bohemicus*). Of these potential actors, only gastropods are preserved as body fossils, and they are concentrated within the upper 50 cm of the main horizon at the site (Foster et al., 2018; Mygatt, 1991), showing that a majority of the decomposers in this Late Jurassic ecosystem have previously gone undetected in the body fossil record. However, this is a minimum estimate of the number of actors at the MMQ. The true diversity was almost certainly far greater as different species can create similar traces, while others may have left no discernable trace in the fossil record (Behrensmeyer, Kidwell & Gastaldo, 2000). Still, the addition of these four potential actors is a significant addition to the paleodiversity of the site, as well as its ecological food web.

Previous reports of invertebrate bioerosion on Morrison vertebrate fossils are confined to a handful of reported specimens from sites in Utah and Wyoming, with many traces attributed to the work of dermestid beetles (Bader, 2008; Bader, Hasiotis & Martin, 2009; Britt, Scheetz & Dangerfield, 2008; Chin & Bishop, 2006; Hasiotis et al., 1999). These studies are invaluable in showing the presence of bone-modifying insects in a geological unit that is devoid of their body fossils. However, isolated specimens may underreport the true abundance of carrion insects and other decomposers in the Morrison ecosystem. Our assemblage-wide survey of bone surface modifications is the first of its kind to be applied to a Morrison site. Our survey draws from thousands of fossils and shows a diversity of trace types and potential actors, as well as the prevalence of these insects, with 16.174% of the total surveyed material preserving at least one of their bioerosion traces. It is difficult to know if the frequencies of bioerosion traces found in this study are typical of attritional mudstone deposits in the Morrison Formation, or if they represent an exemplar scenario at the MMQ. The lack of systematic, assemblage-wide surveys for invertebrate traces on vertebrate bone material in Mesozoic settings is not limited to the Jurassic. Among Cretaceous localities, only De Valais, Apesteguía & Garrido (2012) conducted a systematic, site-wide survey for insect traces in Argentina. However, this was primarily a descriptive study and did not provide any frequencies for insect bone modification at the site, which would have been useful in comparison to those presented here for the MMQ. Additional, site-wide surveys in the Morrison Formation are required to test the overall frequencies of invertebrate traces on vertebrate material across Morrison sites and beyond to other geological units and time periods.

### Residence time for carcasses

Estimating how long animal carcasses were exposed prior to burial in the fossil record can be difficult, as many taphonomic factors alter and distort remains prior to burial (Behrensmeyer, 1978; Behrensmeyer, Kidwell & Gastaldo, 2000; Fisher, 1995). Carrion insects, scavengers and abiotic factors require access to remains in order to modify tissues preserved in the fossil record (in this case, bone). Site taphonomy, weathering stages and entomological succession can offer insight in the estimation of exposure time for vertebrate remains prior to burial.

Foster et al. (2018) provided an extensive taphonomic analysis of the MMQ, examining sedimentology, stratigraphy, bone orientation, and paleontology at the site. Using these data, the authors concluded that the MMQ was formed as an ephemeral pond with slow, attritional deposition in an overbank setting, but without direct connection to a main waterway or perennial standing water at the site. This slow accumulation of clays would require hundreds to thousands of years of deposition to accumulate the 1–2 m of mudstone that is present today (Smith, 1983), and would result in the slow burial of carcasses, especially large sauropod dinosaurs. However, Foster et al. (2018) also noted evidence of trampling and vertebrate bioturbation at the site, which may have facilitated the faster burial of some vertebrate material in this slow-depositional regime, a process that has been observed in modern taphonomic studies (Behrensmeyer, 1978; Behrensmeyer, Kidwell & Gastaldo, 2000). Additionally, the high degree of disarticulation, the lack of element orientation (Foster et al., 2018), and the prevalence of vertebrate feeding traces (Drumheller et al., 2020) indicates extended access to these carcasses by predators/scavengers and trampling.

**Figure 7:**
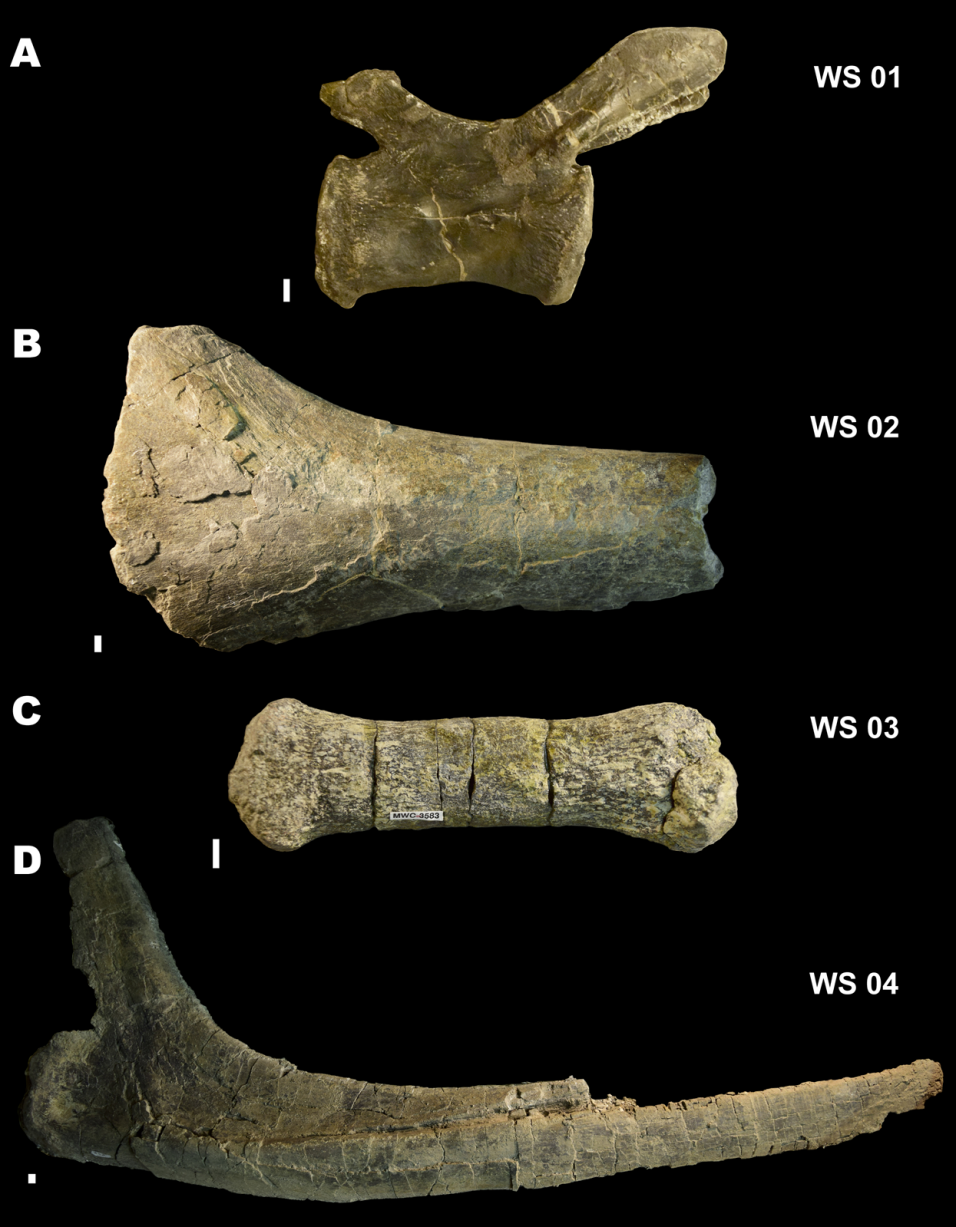
Weathering Stages. Examples of Behrensmeyer’s (1978) weathering stages (WS) in fossil specimens from the Mygatt-Moore Assemblage. (A) WS 01 in MWC 2741, an *Allosaurus* caudal vertebra; (B) WS 02 in MWC 2876, a large Sauropod bone fragment; (C) WS 03 on MWC 3583, a distal caudal vertebra from an *Apatosaurus*; (D) WS 04 in MWC 452, a dorsal rib of an *Apatosaurus*. All scale bars are 10 mm.

The variability in exposure times indicated by site taphonomy is echoed in the variability of weathering stages observed in the fossil material. Foster et al. (2018) reported weathering on 25% of their random subsample survey of MMQ material. We observed similar patterns of weathering in our survey and noted that the stages of weathering ranged from well-preserved, unweathered material (Stage 1 of Behrensmeyer (1978)) to highly weathered material, with multiple layers of periosteum flaked and removed prior to fossilization (Stage 4 of Behrensmeyer (1978)), with the majority of specimens showing better preservation and categorized in Stages 1–2 of Behrensmeyer (1978) (Fig. 7). Based on the actualistic study of weathering stages by Behrensmeyer (1978), these data would indicate residence times at MMQ of a few months, up to 4–6 years for dinosaurian carcasses, and even longer, potentially up to 15 years for the most weathered specimens. However, the climatic and environmental differences between the Jurassic Morrison and the modern ecosystems observed by Behrensmeyer (1978) leave an unknown margin of error around the precision of using these time frames for a long extinct ecosystem. Therefore, these residence time inferences are offered as an estimation only.

The density and diversity of invertebrate traces on bone elements within this dataset shows a rich and thriving invertebrate fauna at the MMQ in the Jurassic. Work on modern dermestid and other insect colonies has allowed forensic entomologists to calculate the sequence and timing of carrion insect modification of vertebrate remains (Backwell et al., 2012; Fernández-Jalvo & Monfort, 2008; Holden, Harris & Timm, 2013; Zanetti, Visciarelli & Centeno, 2014, 2015). Although we have no such clock for the timing of their Jurassic correlates, these studies provide a reference point for estimating the exposure time of dinosaurian carcasses (Laws et al., 1996). Holden, Harris & Timm (2013) used actualistic experiments of dermestid and tenebrionid colonies to calculate their estimate of decomposition time for bones preserved in Rancho La Brea. They found that 17–20 weeks were required from egg laying to pupation into ontogenetic morphs capable of marking bone, of exposure time for the invertebrate-modified distal limb elements. However, the diversity and pervasiveness of invertebrate traces at MMQ is much greater than those reported at La Brea by Holden, Harris & Timm (2013), whose study revealed 20 specimens with insect damage, nearly all epipodial elements, attributable to only two insect actors. Results from this study show a much broader distribution of damage among skeletal elements and higher frequencies of occurrences. Additionally, the morphologies of the identified trace types indicate a higher diversity of actors at MMQ, which when taken with the disparity in sedimentary regimes between the sites, may indicate that longer minimum carcass exposure times were required at the MMQ to generate the preserved invertebrate modified remains, than at La Brea.

### Trace frequency & dinosaurian diversity

The broad similarity of trace types and distributions of other bone surface modifications across taxa in the MMQ provides taphonomic insight into the underlying dinosaurian diversity. The Morrison Formation preserves an unusually high number of sauropods, both in terms of taxonomic diversity and minimum numbers of individuals represented in specific localities (Engelmann, Chure & Fiorillo, 2004; Esker, 2009; Foster, 2003; Whitlock, Trujillo & Hanik, 2018). This has led to a debate on whether this diversity represents a real signal, or if some taphonomic driver is causing the largest-bodied dinosaurs to become concentrated in Morrison sites (Button, Rayfield & Barrett, 2014; Whitlock, Trujillo & Hanik, 2018; Woodruff, 2019). It is tempting to suggest that the heightened number of invertebrate traces and other bone surface modifications on sauropod bones might have been caused by longer residence times necessary to bury such immense sets of remains and may, thus, provide evidence of time averaging directed specifically at sauropod carcasses at MMQ. However, the invertebrate trace fossil frequencies, as well as the distribution of weathering stages, and other bone surface modifications across all MMQ taxa, generally track the taxonomic composition of the site. This suggests that all dinosaurian species present at the site and surveyed in this study were subjected to broadly similar biostratinomic pathways prior to burial. In other words, we find no evidence in this dataset to suggest that any one clade (sauropods) experienced differential taphonomic treatment which resulted in an over-representation in the MMQ assemblage, suggesting that the death assemblage is mimicking the life assemblage in this instance.

The high frequency of invertebrate traces on sauropod remains (78.261% of all invertebrate traces) may have more to do with the disproportionately available bone surface areas compared to other, smaller taxa found at the site (e.g., *Allosaurus*, *Mymoorapelta*, etc.), rather than being a factor of residence time or time averaging. The greater the availability of a resource (in this case: bone surface), the more likely that resource will be utilized. Sauropod remains also preserve the highest frequencies of theropod feeding traces within the examined materials (Drumheller et al., 2020). Sauropod dinosaurs are the largest herbivores in the assemblage and would have been an enormous source of food for both vertebrate and invertebrate actors, both in the number of skeletal elements and their associated sinews and bone surfaces. Additional survey work is required to determine if these results are typical of the Morrison ecosystem, or reflect local conditions at the MMQ.

### Paleoclimatic conditions

**Figure 8:**
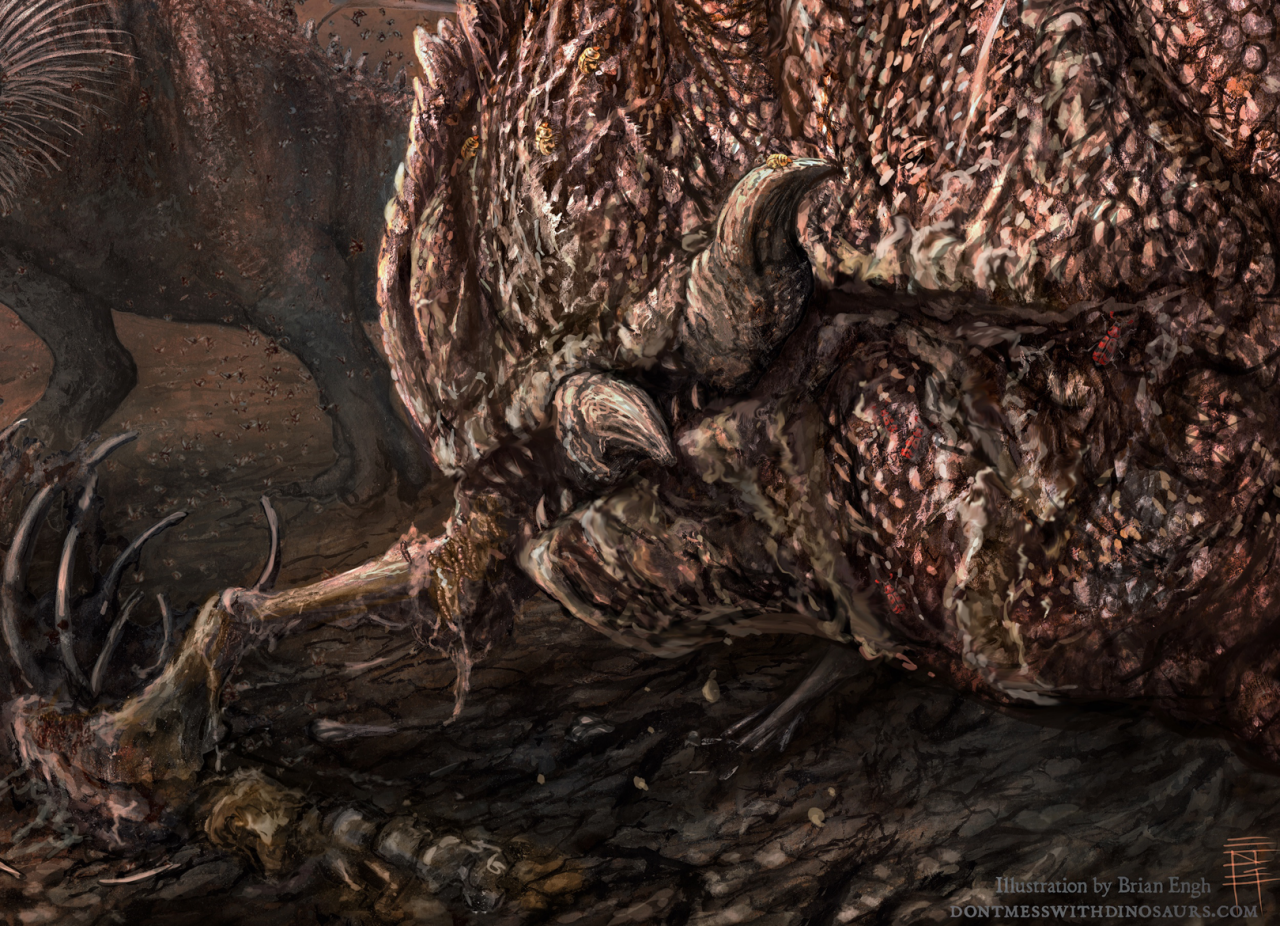
Artist Reconstruction. Dry season at the Mygatt-Moore Quarry showing *Allosaurus* feeding on a rotting, bug-infested carcass. Illustration by Brian Engh, dontmesswithdinosaurs.com (modified with permission from Drumheller et al., 2020).

Invertebrate faunas can also provide insights into paleoclimatic conditions in the fossil record (Elias, 2010; Guiot et al., 1993; Hasiotis, 2004; Holden et al., 2017). Data from experimental studies using dermestid and clerid beetles have demonstrated that these organisms prefer desiccated, though not wholly dry, vertebrate carcasses (Timm, 1982; Zanetti, Visciarelli & Centeno, 2014, 2015) and typically inhabit areas with temperatures above 20 degrees Celcius (Wilches et al., 2016). As dermestids, or behaviorally convergent taxa, are putative actors for the majority of this dataset, we can infer widespread desiccation among the modified remains and a warm, arid paleoclimate, consistent with previous interpretations of ecologically-stressed, resource-poor, drought-induced death assemblages in the Morrison Formation (Foster et al., 2018; Turner & Peterson, 2004) (Fig. 8). However, the presence of gastropods in the assemblage, as both body fossils and potential actors for Trace Type 3, reveals the fluctuation of the local climate at the MMQ and support the presence of periodic wet episodes. Additionally, sedimentological evidence of small (<1 cm^2^) interference ripple marks in the top 50 cm of the horizon and isolated pockets of bioturbation in the lowest meter of the horizon, presumably from dinosaurian faunas churning the smectitic muds like a boggy mire (Foster, 2003, 2014; Foster et al., 2018; McHugh, 2018; McHugh et al., 2018; Mygatt, 1991; Trujillo et al., 2014), support interpretations of the wet/dry seasonality at the MMQ. The disproportionate abundance of Trace Type 1 (dermestid or clerid beetles) compared to Trace Type 3 (gastropods or beetles) in the dataset indicates the dominance of dry conditions during the accumulation of vertebrate carcass remains at the site.

## Conclusions

Invertebrate traces on vertebrate fossil material from the MMQ reveal a hidden and diverse fauna that is not preserved in the body fossil record. These traces are found throughout the site, with 16.174% of the total surveyed assemblage preserving at least one invertebrate trace. Six distinct trace types were identified and, based on size and morphology of the traces, indicate a minimum of four different invertebrate actors. Potential actors for these traces include dermestid beetles, gastropods, an unknown necrophagous insect, and an unknown osteophagous insect. When these traces are examined together with site taphonomy, weathering stages of fossil material, and live insect experiments, they support predominantly dry conditions, and long residence times for dinosaurian carcasses at the MMQ, ranging from five months up to six years or more. Comparison to other sites in the Jurassic is difficult due to the lack of similar comprehensive surveys of bone surface modifications on bulk collected specimens. Given the small size of some of these traces, and the tendency to collect better preserved, more taxonomically informative elements for permanent curation (Drumheller et al., 2020), they could easily be overlooked by researchers not specifically looking for them. We hypothesize that invertebrate traces on vertebrate bone are more common in the Jurassic Morrison Formation than previously realized. However, more work is needed to determine of the invertebrate modified fossils from the MMQ represent an exemplar decomposer community or provide evidence for the underreporting of invertebrate traces from other fossil sites.

## Supplemental Information

10.7717/peerj.9510/supp-1 Supplemental Information 1 Modified Materials. List of specimens surveyed that preserve bone surface modifications.

10.7717/peerj.9510/supp-2 Supplemental Information 2 Examined Specimens. List of fossil specimens from Mygatt-Moore Quarry surveyed in this study.

## Institutional abbreviation

MWC: Museums of Western Colorado

## Additional Information and Declarations

### Field Study Permissions

The following information was supplied relating to field study approvals (i.e., approving body and any reference numbers): Field collection was approved by the United States Bureau of Land Management (paleontological permit # COC76588).

### Data Availability

The following information was supplied regarding data availability: All examined materials are available in Tables S1 and S2.
